# Electronic cleansing in computed tomography colonography using AT layer identification with integration of gradient directional second derivative and material fraction model

**DOI:** 10.1186/s12880-017-0224-6

**Published:** 2017-09-04

**Authors:** Krisorn Chunhapongpipat, Ratinan Boonklurb, Bundit Chaopathomkul, Sirod Sirisup, Rajalida Lipikorn

**Affiliations:** 1 Machine Intelligence and Multimedia Information Technology laboratory (MIMIT Lab), Department of Mathematics and Computer Science, Faculty of Science, Chulalongkorn University, Payathai Road, Bangkok, 10330 Thailand; 2 Department of Mathematics and Computer Science, Faculty of Science, Chulalongkorn University, Payathai Road, Bangkok, 10330 Thailand; 3 Department of Radiology, Faculty of Medicine Chulalongkorn University, King Chulalongkorn Memorial Hospit, Rama 4 Road, Bangkok, 10330 Thailand; 4 Large-Scale Simulation Research Laboratory, National Electronics and Computer Technology Center, 112 Thailand Science Park, Pathumthani, 12120 Thailand

**Keywords:** Electronic colon cleansing, Partial volume effect, Pseudo-enhancement effect, Computed tomography colonography

## Abstract

**Background:**

In computed tomography colonography images, electronic cleansing (EC) is applied to remove opacified residual materials, called fecal-tagging materials (FTM), using positive-contrast tagging agents and laxative to facilitate polyp detection.

**Methods:**

The proposed EC, *EC*_*prop*_, integrates the gradient directional second derivative into material fraction model to preserve submerged soft tissue (ST) under FTM. Three-material fraction model is used to remove FTM and artifacts at air-tagging (AT) layers and T-junctions where air, ST, and FTM material meet simultaneously. Moreover, the proposed AT layer identification is used to distinguish AT layers from air-tissue-tagging (ATT) layers in order to preserve ATT layers during cleansing. The clinical evaluation on 467 3-Dimensional band view images was conducted by the abdominal radiologist using four grading levels of cleansing quality with five causes of low quality EC. The amount of the remaining artifacts at T-junctions was approximated from the results of *EC*_*prop*_. The results from *EC*_*prop*_ were compared with the results from syngo.via Client 3.0 Software, *EC*_*syngo*_, and the fast three-material modeling, *EC*_*prev*_, using the preference of the radiologist. Two-tailed paired Wilcoxon signed rank test is used to indicate statistical significance.

**Results:**

The average grade on cleansing quality is 2.89 out of 4. The artifacts at T-junctions from 86.94*%* of the test images can be removed, whereas artifacts at T-junctions from only 13.06*%* of the test images cannot be removed. For 13.06*%* of the test images, the results from *EC*_*prop*_ are more preferable to the results from *EC*_*syngo*_ (*p*<0.008). For all the test images, the results from *EC*_*prop*_ are more preferable to the results from *EC*_*prev*_ (*p*<0.001). Finally, the visual assessment shows that *EC*_*prop*_ can preserve ATT layers, submerged polyps and folds while *EC*_*prev*_ can preserve only submerged folds but fails to preserve ATT layers.

**Conclusion:**

From our implementation, *EC*_*prop*_ can improve the performance of the existing EC, such that it can preserve ST, especially ATT layers and remove the artifacts at T-junctions which have never been proposed by any other methods before.

## Background

Virtual colonoscopy (VC) or computed tomography colonography (CTC) is one of the most acceptable techniques for non-invasive screening of colon cancer [1–5]. Colon cancer is the second common cause of cancer death in the United States [6], whereas the second cause of cancer death in Thailand is colon and rectum cancer [7]. With the advances in medical imaging and computer technologies, CTC can simulate the colonoscopy screening for polyps from 3-Dimensional (3D) fly-through virtual colon surface reconstruction of the abdominal part of human [8, 9]. Although a colonoscopy is the gold standard for polyp screening [5], but CTC is more preferable because of safety, cost and noninvasive procedure [10–12].

To enhance the ability of polyp detection while performing 3D fly-through in virtual colonoscopy, electronic cleansing (EC) [13–21] is used to remove the residual colonic materials in CTC data. The oral contrast agents [22] are given to a patient to lighten the residual colonic materials, called fecal-tagging materials (FTM), in order to distinguish them from soft tissue (ST). For the accuracy of diagnosis of polyp screening from the radiologist, foods are limited and laxative is used to clear up the colonic lumen for clear images. However, the research [23] on colonography without cathartic or laxative was conducted according to low acceptance rate of both optical and typical cathartic virtual colonoscopy [24]. The results [23] show that it can almost compete with conventional optical colonoscopy for detecting asymptomatic adults with adenomas ≥10 mm. In contrast, laxative-free CTC performance declines for smaller lesions [23]. Moreover, there is no missing for colorectal cancer detection [25] when both cathartic and oral contrast are used together. Eventaully, CTC data with both cathartic and oral contrast are still practical in many health care institutes, such as in Japan [26], UW Health in USA [27], University of California, San Francisco, in USA [28], Bumrungrad International Hospital, Bangkok, in Thailand [29], King Chulalongkorn Memorial hospital, Bangkok, in Thailand, etc.

Computed tomography (CT) attenuations of ST around FTM are irregularly higher than their regular range because of pseudo-enhancement (PEH) effect which is the effect of FTM. CT attenuations of PEH ST around FTM can be reduced by using PEH correction algorithms [30–32]. In general, these methods [30–32] approximate the PEH effect and then subtract it from CT attenuations of the FTM and their vicinity to obtain the PEH correction results.

For the artifact layer between air and FTM, it is called partial volume (PV) effect layer [18] or AT layer [13] which is the local nonlinear volume averaging of CT attenuations between two components. Wang et al. [18] proposed the improved maximum a *posteriori* expectation-maximization (MAP-EM) image segmentation algorithm which approximates tissue mixture percentages in a voxel with statistical model parameters for tissue distribution. This method removes PV effect and prevents a chance of incomplete and overcomplete cleansing in EC. The structural response [13] with rut and cup structures is used to preserve PEH ST voxels between FTM and submerged polyps or folds. The local roughness response [13] is used to determine whether a voxel belongs to an AT or an ATT layer. An AT layer is an artifact between air and FTM, whereas an ATT layer is a thin ST layer between air and FTM. These layers are similar to each other by their gradient magnitudes and CT attenuations. The results of the structural analysis (SA) level set [13] can preserve an ATT layer and thin folds between FTM, submerged folds and polyps. Alternatively, the boundary between tagged pool and ATT layers might be extracted using method from Chen et al. [33]. Lee et al. [15] integrated rut-shape structural response [13] with material fraction model [34] to preserve folds. CT attenuations of a layer between air and FTM and CT attenuations of an FTM region are replaced by CT attenuations of pure material of air. CT attenuations of a layer between ST and FTM are replaced by the result from integrating the rut-shape structural response [13] into the material fraction model between ST and FTM. The results of Lee et al. [15] show that their proposed method can preserve folds better than the existing method [34] which is the material transition between two materials that is scaling and rotation invariant. Furthermore, other translation, scaling, rotation invariant features and noise suppression can be found in methods from Zhang et al. [35] and Chen et al. [36], respectively. Next, Serlie et al. [20] proposed three-material fractions approximation from the scale-invariant three-material model to remove the artifacts at the T-junctions where air, ST, and FTM meet simultaneously. Later on, the barycentric coordinates of a triangle from three pairs of two material transitions is used to speed up the method of three-material fractions approximation [20] by the proposed method from Lee et al. [16]. The integration of linear combination of rut-shape structural response [13] and three material fractions was proposed by Lee et al. [16]. The results from this method [16] contain no artifact at the junction of three materials and the folds can also be preserved.

Although, there are state-of-the-art methods [13, 15, 16, 18, 20, 34] that can solve several issues in EC recently, none of these methods can solve all of the issues as shown in Table 1. Thus, the aim of this paper is to propose EC in CTC for patient preparation with both cathartic and oral contrast that can preserve PEH ST voxels and ATT layers and can remove AT layers and artifacts at T-junctions. Recently, there is no theoretical support on artifact removal at T-junction in the SA level set method [13], and ATT layer preservation in other existing methods [15, 16].

**Table 1:** Several issues were solved by the existing ECs

Previous work	PEH	PVE	T-junction	AT and ATT
Wang et al. [18]	-	✓	-	-
Serlie et al. [34]	-	✓	-	-
Serlie et al. [20]	-	✓	✓	-
Cai et al. [13]	✓	✓	-	✓
Lee et al. [15]	✓	✓	-	-
Lee et al. [16]	✓	✓	✓	-

## Methods

This paper proposes the EC method, *EC*_*prop*_, in CTC. Three data sets of CTC images were used to evaluate the proposed method. The cleansing quality evaluation was performed by the abdominal radiologist. Moreover, the comparisons between results from *EC*_*prop*_ and those from the commercial software and the another method which are the syngo.via Client 3.0 commercial Software from Siemens CT scanner and the fast three-material model [16] were also performed by the radiologist.

### CTC data

CTC data sets from Walter Reed Army Medical Center (WRAMC) and King Chulalongkorn Memorial Hospital were used as the test data, whereas, the training set is the CTC data set which was acquired from Philips CT scanner, Brilliance 64 model, with the same patient preparation as Pickhardt et al. [37]. Each scan contains a set of approximately 600 slices of images size 512×512 pixels with the spatial resolution of approximately 0.66 mm. × 0.66 mm. × 0.7 mm, 197mA X-ray tube current, and a voltage of 120 kVp. Among these data sets, five cases (patients) of CTC data were randomly selected from WRAMC which could be downloaded from the National Institutes of Health that provides a CT colonography database with complete associated colonoscopy findings (imaging.nci.nih.gov) and ten cases of CTC data were randomly selected from King Chulalongkorn Memorial Hospital as the test data. Both prone and supine positions from CTC data sets were used for evaluation.

### The proposed EC method

The proposed algorithm consists of the following processes:

1. Remove the lung and bone volumes from CTC data.
2. Detect colonic lumen.
3. Detect ambiguous layers which are AT and ATT layers.
4. Identify AT layers from ambiguous layers.
5. Detect STT layers which are the boundaries between FTM and ST by removing AT layers from the boundaries of FTM.
6. Approximate material fractions of three materials.
7. Approximate gradient directional second derivative (GDSD).
8. Integrate GDSD into material fraction model.
9. Enhanace colonic wall.

#### Lung and bone removal

**Fig. 1:**
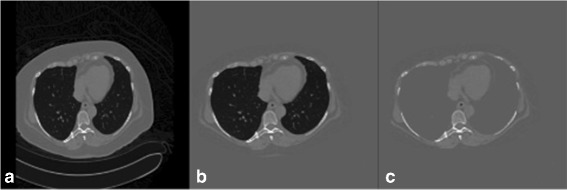
**a** the original CTC image. **b** the result from outside air removal. **c** the result from lung removal

In order to detect colonic lumen, air and bones outside colonic lumen are needed to be removed because CT attenuations of air and FTM inside colonic lumen are similar to CT attenuations of air and bones outside colonic lumen. Thus, the lungs and bones must be detected and removed by using the following procedure:

1. At the beginning, air is divided into two components. The first component is the air outside the body while the second component is the air inside the body. The location of the outside air can be detected using region growing on a binary image, where seed voxels are located at the image boundary. To obtain all voxels of outside air, the seed voxels grow to all voxels with CT attenuations less than −100 HU where −100 HU is the lowest CT attenuation of ST according to Table 2. For all outside air voxels in a binary image, CT attenuations of voxels are changed to −100 HU as shown in Fig. 1 b.

**Table 2:** The observed standard CT attenuations of each material in HU [13]

Component	Minimum	Maximum
lumen air	-1000 HU	-800 HU
ST	-100 HU	100 HU
FTM	200 HU	1400 HU
AT Layer	-800 HU	600 HU
2. The remaining inside air is further divided into two components which are air inside the lungs as the first component and air inside colonic lumen as the second component. To locate the lung locations, CTC data of a patient are divided into three segments. The first segment is the first 10 slides while the second segment is the last 10 slides. The numbers of inside air voxels with CT attenuations less than −100 HU in the first segment and the second segment are compared. A segment with larger number of inside air voxels is considered as the segment with lungs. The seed voxels are randomly selected and region growing is used to obtain the lung locations by having the seed voxels grow to all voxels with CT attenuations less than −100 HU. Then the lungs are removed by changing CT attenuations of lung voxels to −100 HU as shown in Fig. 1 c.
3. The last step is to locate the bones. The seed voxels for region growing are placed at voxels with CT attenuations higher than 200 HU in the same slides of lung locations. This placement also includes the locations of rib around the lungs. In order to obtain bone locations, the seed voxels grow to voxels with CT attenuations greater than 200 HU. Then the bones are removed by changing CT attenuations of bone voxels to −100 HU.

The above procedure transforms CT attenuations of the lungs and the bones into the lower bound of ST to facilitate the next step. The following step is to approximate the location of the colon using CT attenuations of air and FTM.

#### Colonic lumen detection

**Fig. 2:**
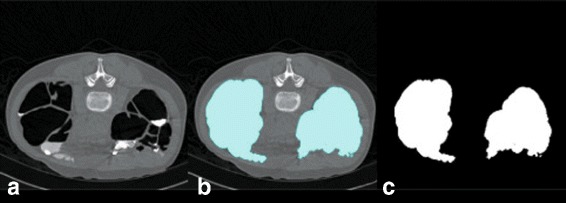
**a** the original CTC image. **b** the locations of colonic lumen in a CTC image. **c** the locations of colonic lumen which are represented by white pixels in a binary image

The locations of colonic lumen are detected by merging air and FTM together as shown in Fig. 2. In order to merge air and FTM, first the locations of air and FTM are identified at the voxels with CT attenuations lower than −600 HU and higher than 200 HU [13], respectively. The images of air and FTM are then converted to binary images. Next, morphological dilation with spherical structure element of radius equal to 3 is performed to merge air and FTM locations where the thinkness of PV effect layer is three voxels. To reduce computational complexity, all computations are performed only inside the colonic lumen.

#### Ambiguous layer detection

**Fig. 3:**
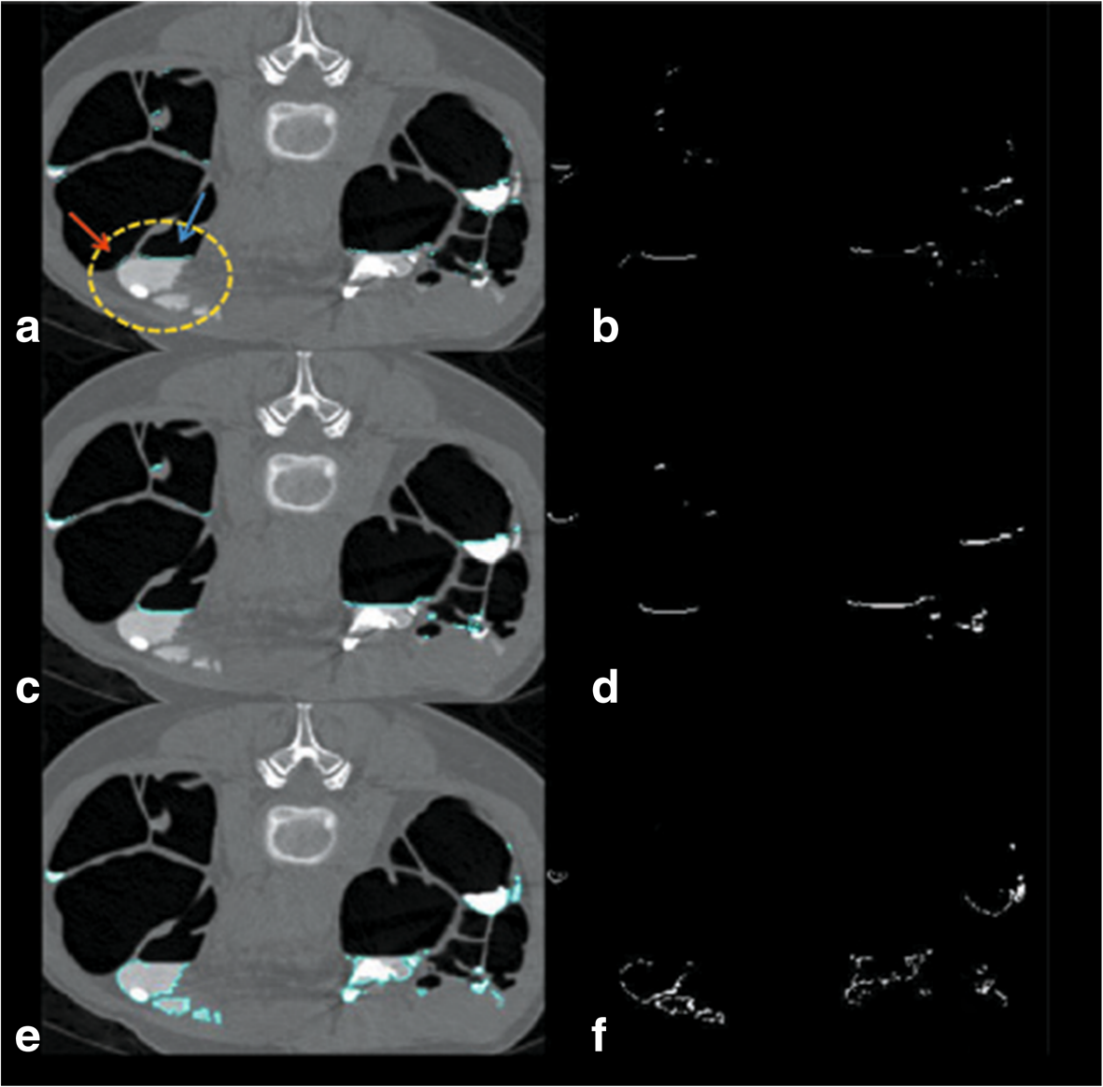
**a** a CTC image with the ambiguous layers. The yellow dash circle contains an AT layer and an ATT layer where an AT layer is pointed by the blue arrow while an ATT layer is pointed by the red arrow. **b** the locations of ambiguous layers in a binary image. **c** the locations of AT layers in a CTC image. **d** the locations of AT layers in a binary image. **e** the locations of STT layers in a CTC image. **f** the locations of STT layers in a binary image

An ambiguous layer is a thin layer between air and FTM which could be either an AT layer or an ATT layer [13]. In colonic lumen, there are three types of edges; the first type is the edge between air and ST, the second type is the edge between air and FTM, and the last type is the edge between ST and FTM. In order to obtain the edge between air and ST, 26 neighbors of each voxel of the first and the second edge types are used. If there is any FTM voxel in the neighbors of any considered edge voxel, that edge voxel is removed. The remaining voxels are the edge between air and ST. Thus, the ambiguous layers can be obtained by removing the edge between air and ST from the boundary of air in colonic lumen as shown in Fig. 3 a and b where Fig. 3 a shows ambiguous layers in a CTC image while Fig. 3 b shows the locations of ambiguous layers in a binary image.

#### AT layer identification

In order to identify an AT layer, the CT scanner table must be at the bottom of a CTC image as shown in Fig. 2 a. Thus, FTM is now on top of an ATT layer.



Although, the CT scanner table is at the bottom of a CTC image, but an AT layer does not necessary have to be on top of FTM because it could be an ATT layer instead as shown in the yellow dash circle of Fig. 3 a and b.

In the yellow dash circle, FTM lies against two ambiguous layers. The one pointed by the red arrow is an ATT layer while another one pointed by the blue arrow is an AT layer.

To distinguish an AT layer from an ATT layer, the numbers of connectivities in horizontal and vertical directions from voxels in an ambiguous layer to voxels in FTM are compared using Algorithm 1. Thus, an ATT layer can be eliminated from ambiguous layers as follows:

1. Label FTM regions in a CTC image.
2. Label all ambiguous layers in a CTC image.
3. Compare the numbers of connectivities between horizontal and vertical directions from voxels in the *j* ^*th*^ ambiguous layer to the *i* ^*th*^ FTM in a CTC image by using Algorithm. 1 where *r* and *s* are the minimum and maximum indice of ambiguous layers that lie against the *i* ^*th*^ FTM and *r*≤*j*≤*s*.

To measure the connectivity, the leaping distance of five voxels [15, 16] is used. For any voxel of the *j* ^*th*^ ambiguous layer, if the neighbor voxels in leaping distance are in the *i* ^*th*^ FTM, the connectivity of that specific direction is counted. The horizontal direction is divided into left and right directions. The vertical direction uses only downward direction. Because an AT layer normally lies on FTM since the CT scanner table is at the bottom of a CTC image.

For the case of an AT layer, the number of connectivities in vertical direction is higher than that in horizontal direction because an AT layer normally lies on the adjacent FTM. For the case of an ATT layer, the number of connectivities in horizontal direction is higher than that in vertical direction because an ATT layer is normally attached to the adjacent FTM along the bend of tagged pool. Thus, if the number of connectivities in horizontal direction is higher than that in vertical direction, the *j* ^*th*^ ambiguous layer must be removed. Finally, the remaining labeled ambiguous layers are AT layers as shown in Fig. 3 c and d.

#### STT layer detection

An AT layer is the interface between air and FTM, whereas a STT layer is the edge between ST and FTM that also includes an ATT layer. The vicinity of FTM contains both STT layers and AT layers. In order to identify STT layers, all AT layers are removed from the vicinity of FTM and the remainning layers are STT layer as shown in Fig. 3 e and f.

#### Material fraction approximation

An AT layer is used to find two material fractions from material transition between air and FTM. A STT layer is used to find two material fractions from material transition between ST and FTM. Thus, two material fractions from material transition between two materials can be approximated according to the following description. The transition between two materials [15, 16, 20, 34] is modeled from the result, *G*, of the convolution between the unit-step function, *u*, and Gaussian, *g*, with standard deviation, *σ*, where

1 $$\begin{array}{*{20}l}  G(x;\sigma) &= u(x)*g(x;\sigma)=\frac{1}{2} + \frac{1}{2}\text{erf}\left(\frac{x}{\sigma\sqrt{2}}\right) \end{array} $$

2 $$\begin{array}{*{20}l} u(x) &= \left\{\begin{array}{ll} 0, &x<0, \\ 1, &x\geq{0}, \end{array}\right.  \end{array} $$

3 $$\begin{array}{*{20}l} g(x,\sigma)&= \frac{1}{\sigma\sqrt{2\pi}}\text{exp}\left(\frac{-x^{2}}{2\sigma^{2}}\right) \end{array} $$

and

4 $$ \text{erf}(x) = \frac{2}{\sqrt{\pi}}\int^{x}_{0}\text{exp}\left(-t^{2}\right)dt.  $$

The first derivatives along gradient direction, *I*_*w*_, and CT attenuations, *I*, are collected from the edge between material of *a* and *b*. The CT attenuation is represented by

5 $$ I\left(\omega;\sigma_{\omega}\right)\equiv\left(H_{ab}-L_{ab}\right)G\left(\omega;\sigma_{\omega}\right)+L_{ab}  $$

and the first derivative along gradient direction is represented by

6 $$ I_{\omega}\left(\omega;\sigma_{\omega}\right)\equiv\left(H_{ab}-L_{ab}\right)g\left(\omega;\sigma_{\omega}\right)  $$

where *H*_*ab*_ and *L*_*ab*_ are the bases of transition in higher and lower sides of an edge between *a* and *b*, respectively. *ω* is the gradient direction and *σ*_*ω*_ is the scale of Gaussian along *ω* direction.

The first derivative along gradient direction is normalized by the scaled gradient magnitude *σ*_*ω*_ *I*_*ω*_ to yeild scale invariance *σ*_*ω*_ *I*_*ω*_. The function between *σ*_*ω*_ *I*_*ω*_ and *I* is modeled by:

7 $$ \sigma_{\omega}I_{\omega}=\left(H_{ab}-L_{ab}\right)arch\left(\frac{I-L_{ab}}{H_{ab}-L_{ab}}\right),  $$

where

8 $$ \begin{aligned} arch(x) &\equiv\sigma_{\omega}g\left(G^{-1}(x;\sigma_{\omega});\sigma_{\omega}\right)\quad\\ &=\frac{1}{\sqrt{2\pi}}\text{exp}\left(-\left(\text{erf}^{-1}(2x-1)\right)^{2}\right), \end{aligned}   $$

for *x*∈ [ 0,1].

**Fig. 4:**
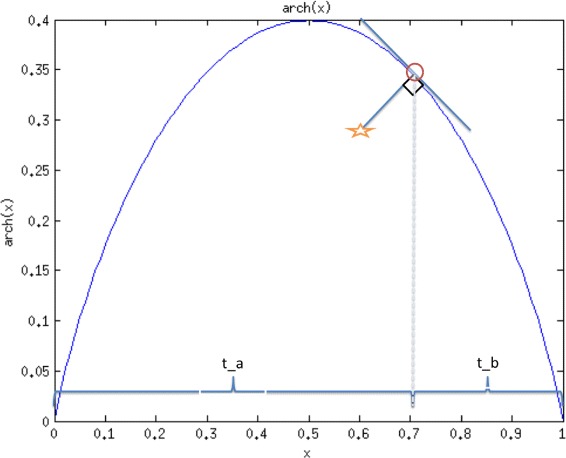
The material transition between *a* and *b*. {*I*,*θ* *σ*_*ω*_ *I*_*ω*_} is represented by star. {*I* ^′^,*θ* *σ*_*ω*_ *I* *ω*′} at the intersection of orthogonal line to tangent line is the red circle point of *arch*(*x*). {*I* ^′^,*θ* *σ*_*ω*_ *I* *ω*′} on *arch*(*x*) is used to find two material fractions *t*_*a*_ and *t*_*b*_

The material fractions between materials *a* and *b* are estimated from the relation of {*I*,*θ* *σ*_*ω*_ *I* *ω*} where *θ* is a factor to make, *θ* *σ*_*ω*_ *I*_*ω*_, noise invariance. After {*I*,*θ* *σ*_*ω*_ *I*_*ω*_} is obtained, the orthogonal projection is used to find two material fractions of {*I*,*θ* *σ*_*ω*_ *I*_*ω*_} onto the closest point {*I* ^′^,*θ* *σ*_*ω*_ *I* *ω*′} of *arch*(*x*) in Eq. (8). In Fig. 4, two material fractions *t*_*a*_ and *t*_*b*_ from the closest point {*I* ^′^,*θ* *σ*_*ω*_ *I* *ω*′} at the red circle between *L*_*ab*_ and *H*_*ab*_ can be obtained by

9 $$ t_{b}=\frac{H_{ab}-I'}{H_{ab}-L_{ab}},t_{a}=\frac{I'-L_{ab}}{H_{ab}-L_{ab}}=1-t_{b},  $$

Let

- $t_{L_{TT}}$ and $t_{H_{TT}}$ be material fractions from material transition between ST and FTM,
- $t_{L_{TA}}$ and $t_{H_{TA}}$ be material fractions from material transition between air and FTM,
- $t_{L_{SA}}$ and $t_{H_{SA}}$ be material fractions from material transition between ST and air,
- *L*_*TT*_ and *H*_*TT*_ be ST and FTM bases of material transition between ST and FTM,
- *L*_*TA*_ and *H*_*TA*_ be air and FTM bases of material transition between air and FTM,
- *L*_*SA*_ and *H*_*SA*_ be ST and air bases of material transition between ST and air.

Thus, three material fractions of air, ST, and FTM in a voxel can be approximated [16] by

10 $$ t_{air}=\frac{t_{L_{SA}}+t_{L_{TA}}}{3},  $$

11 $$ t_{ST} =\frac{t_{H_{SA}}+t_{L_{TT}}}{3},  $$

12 $$ t_{TR} =\frac{t_{H_{TA}}+t_{H_{TT}}}{3}= 1-t_{air} - t_{ST},  $$

where *t*_*air*_, *t*_*ST*_, *t*_*TR*_ represent fraction of air, fraction of ST, and fraction of FTM in a voxel, respectively. Then, $t_{L_{SA}}$ and $t_{H_{SA}}$ can be approximated using *L*_*TA*_ and *L*_*TT*_ as bases of material transition between air and ST.

#### GDSD approximation

GDSD, *f*_*ωω*_, can be approximated from the convolution result, *f*, between CTC data and Gaussian function with scale *σ*=1 as

13 $$ \begin{aligned} f_{\omega \omega} &= \frac{1}{| \nabla{f} |^{2} }\left(f_{x}f_{x}f_{xx} + f_{y}f_{y}f_{yy}+\right.\\ &f_{z}f_{z}f_{zz} + 2f_{x}f_{y}f_{xy} + \\ &\left.2f_{x}f_{z}f_{xz}+ 2f_{y}f_{z}f_{yz}\right), \end{aligned}   $$

where $|\nabla f |=\sqrt {f_{x}^{2}+f_{y}^{2}+f_{z}^{2}}$, *f*_A_ and *f*_AB_ are the first and second derivatives of *f* and −1<*f*_*ωω*_<1.

**Fig. 5:**
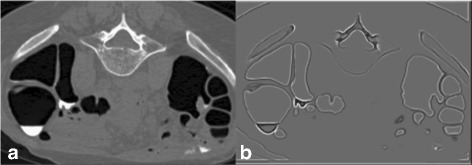
**a** a CTC image. **b** the gradient directional second derivative of CTC image **a**. White line is high positive of GDSD. ST at Vicinity of FTM is in the white line. Black line is high negative of GDSD. FTM is in the black line

The magnitude of GDSD is high at vicinity of an edge and is getting lower when it is further away from an edge. Normally, there are two sides of edge vicinity where CT attenuations of one side are higher than that of the other side. The GDSD is positive on the lower side. This positive side contains ST and AT layers around FTM as shown in Fig. 5 b. However, CT attenuations of ST around FTM could be higher than 100 HU [30] due to PEH effect. Thus, CT attenuations of these voxels must be corrected to be in the ST range.

The GDSD is integrated into material fraction model of material transition between ST and FTM to take care of PEH effect on ST around FTM as described in the next section.

#### GDSD material fraction EC

CT attenuation, *I*, can be modeled [15] using the linear combination of two material fractions from material transition between *a* and *b* as follows:

14 $$  I= t_{L_{ab}}\cdot L_{ab} + t_{H_{ab}}\cdot H_{ab}.  $$

Thus, CT attenuations of voxels in an STT layer around the vicinity of FTM can be calculated using Eq. (14) as follows:

15 $$  I_{TT} = t_{L_{TT}} \cdot L_{TT} + t_{H_{TT}} \cdot H_{TT}.  $$

However, there are PEH soft-tissue voxels in the vicinity of FTM whose CT attenuations are higher than their standard range. Thus, only Eq. (14) is not sufficient to solve PEH problem.

CT attenuations of STT layers around FTM are changed by using the proposed integration of GDSD into the linear combination of two material fractions from material transition between ST and FTM as follows:

16 $$ \begin{aligned} I_{\text{peh}} & = t_{L_{TT}}\cdot L_{TT} \\ &\quad+ t_{H_{TT}}\cdot \left\{f_{{\omega \omega}}\cdot L_{TA} + \left(1-f_{{\omega \omega}}\right)\cdot H_{TA}\right\}, \end{aligned}   $$

where *f*_*ωω*_>0 and *I*>100.

For CT attenuations of FTM, AT layers, and artifacts at T-junctions, they are changed by using the linear combination of three material fractions [16] as follows:

17 $$ I_{T_{junction}}=t_{air}\cdot L_{TA} + t_{ST}\cdot L_{TT} + t_{TR}\cdot L_{TA}.   $$

#### Colonic wall enhancement

In order to smooth colonic wall after applying the proposed EC method, the edges between air and colonic wall that are used to be the edges between FTM and colonic wall are smoothed using Gaussian smooth function with specific scale *σ*=0.5.

**Fig. 6:**
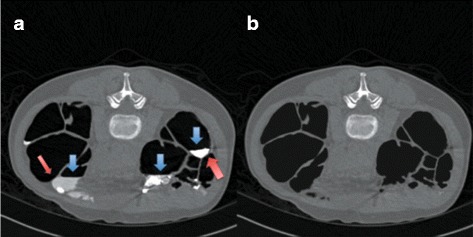
**a** a CTC image. Blue arrows point at AT layers that are removed at the same time as FTM. Red arrows point at ATT layers that are preserved from EC. **b** the final result from our proposed EC method

Figure 6 shows an example of the final result after applying the proposed EC. The final result shows that the AT layer and artifacts at T-junctions are removed while the ATT layers are preserved.

### Clinical evaluation

The clinical evaluation was performed by the abdominal radiologist with clinical experience over 10 years. Three dimensional volume-rendered images of panoramic endoluminal view, called band view [16], were generated by VitreaCore fX Version 6.5.5036.1 software from Toshiba CT scanner which is currently used at King Chulalongkorn Memorial hospital. The evaluation was performed on 467 of band view images and axial images which were collected from the EC results of the test data set.

**Fig. 7:**
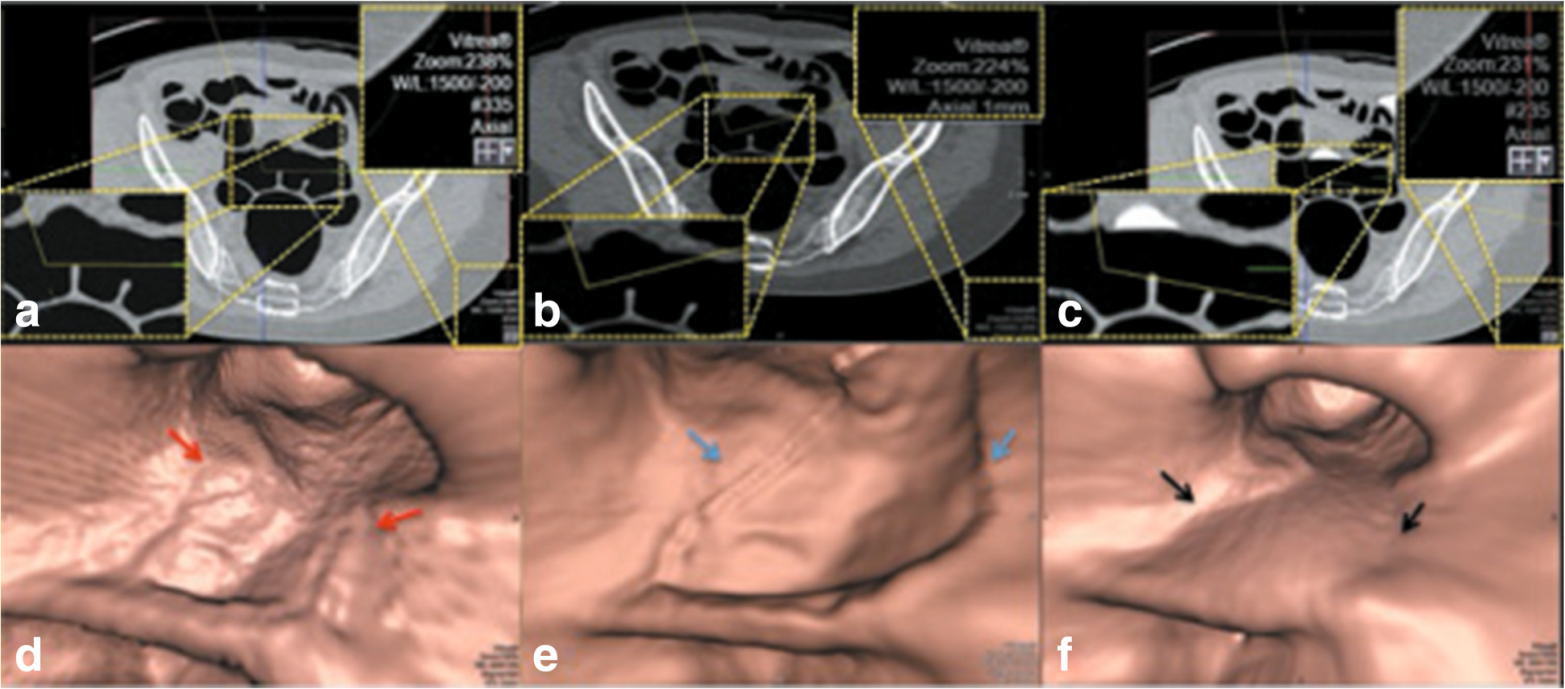
**a** the axial CTC image of the EC result from *EC*_*prop*_. **b** the axial CTC image of the EC result from *EC*_*syngo*_. **c** the axial CTC image without applying any EC. **d** the 3-D band view image of **a**. **e** the 3-D band view image of **b**. **f** the 3-D band view image of **c**

**Fig. 8:**
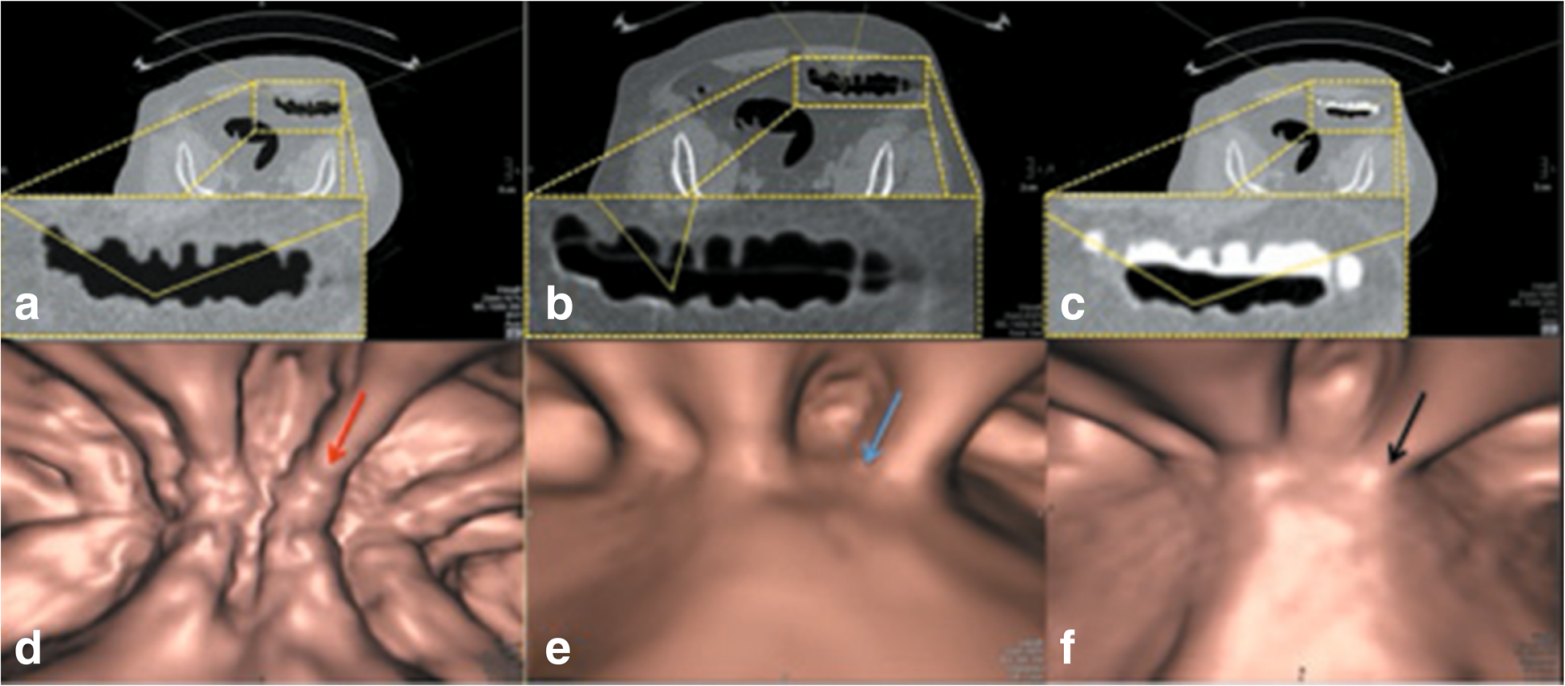
**a** the axial CTC image of the EC result from *EC*_*prop*_. **b** the axial CTC image of the EC result from *EC*_*syngo*_. **c** the axial CTC image without applying any EC. **d** the 3-D band view image of **a**. **e** the 3-D band view image of **b**. **f** the 3-D band view image of **c**

**Fig. 9:**
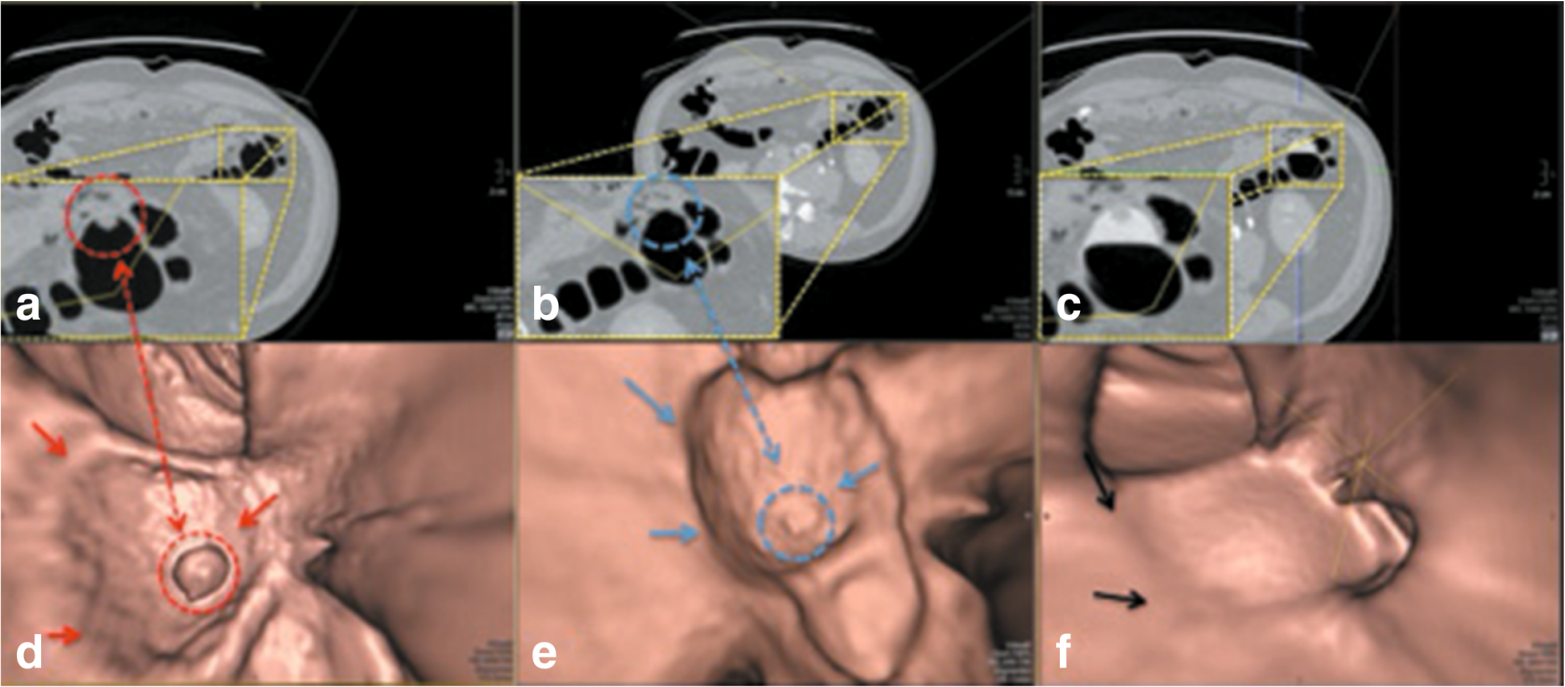
**a** the axial CTC image of the EC result from *EC*_*prop*_. **b** the axial CTC image of the EC result from *EC*_*syngo*_. **c** the axial CTC image without applying any EC. **d** the 3-D band view image of **a**. **e** the 3-D band view image of **b**. **f** the 3-D band view image of **c**

Figures 7, 8, 9, 11, and 12 are some samples of the images where the axial images are shown on the top rows of the figures and the band view images are shown on the bottom rows of the figures. The left columns of the figures display the EC results from *EC*_*prop*_ while the middle columns of the figures display the EC results from *EC*_*syngo*_ or *EC*_*prev*_ [16] where *EC*_*syngo*_ is the commercial virtual colonoscopy software, syngo.via Client 3.0 from Siemens CT Scanner which is currently used at King Chulalongkorn Memorial hospital and *EC*_*prev*_ is the fast three-material modeling EC method [16], respectively. The middle columns of Figs. 7, 8, and 9 are the EC results from *EC*_*syngo*_, whereas the middle columns of Figs. 11 and 12 are the EC results from *EC*_*prev*_. The right columns display the axial images and the band view images before applying EC methods. All the axial images use CT attenuations from −200 to 1500 HU as shown in the window on the top right of Fig. 7 a, b, and c.

The clinical evaluation was performed using four grading levels for cleansing quality with five causes of low quality EC [15]. The grading scheme in cleansing quality evaluation with five causes for low quality EC from Lee et al. [15] was used with band view images and axial images. The cleansing quality is divided into four grades which are inadequate, moderate, good, and excellent as shown in Table 3. The causes of low quality indicate the reason for grading the cleansing quality as shown in Table 4. For the lowest grade, the inadequate grade indicates incomplete FTM removal. For the rest of cleansing quality grades, they indicate complete FTM removal (100% of cleansing). For incomplete and complete FTM removal, they are differentiated by using the axial images of the EC results. For the excellent grade, it means that no artifact can be found in a result after cleansing. For the moderate and good grades, they are differentiated by cleansing quality.

**Table 3:** Grading scheme in cleansing quality evaluation

Cleansing quality grade			
1	Inadequate	0/467	0.00%
2	Moderate	135/467	28.91%
3	Good	247/467	52.89%
4	Excellent	85/467	18.20%

**Table 4:** Five causes of low quality EC

Reason of low quality EC		
Artifacts at T-junction	326/467	69.81%
Inhomogeneous tagging	0/467	0.00%
Collapsed area	0/467	0.00%
Image noise	0/467	0.00%
Incomplete EC	0/467	0.00%

If the artifacts at T-junctions are reported by the radiologist, we need to measure how much the artifacts at T-junctions still remain. The amount of the remaining artifacts at T-junctions is approximated from the result images of *EC*_*prop*_ by the radiologist. If the radiologist cannot identify any artifact at T-junctions, it means that *EC*_*prop*_ is successful. On the other hand, if the radiologist can identify the remaining artifacts in the band view image, the amount of the remaining artifacts at T-junctions is approximated. The amount of the remaining artifact is divided into eight intervals from the experience of the radiologist as shown in Table 7. From these eight intervals, 100% means that the whole artifact is remained while 0% means that the artifact is removed completely.

Furthermore, the comparison on cleansing results with 100% remaining artifacts at T-junction between *EC*_*prop*_ and *EC*_*syngo*_ is performed to measure the cleansing quality of these two methods. The preference is divided into three scales as shown in Table 6 where scales 1,2 and 3 mean slightly better, better and much better, respectively.

In order to perform the comparison between *EC*_*prop*_ and *EC*_*prev*_, the radiologist defined seven scales of preference as shown in Table 7 where scales 1, 2, and 3 mean *EC*_*prop*_ [16] is slightly better, better, and much better, respectively. In contrast, preference scales −1, −2, and −3 mean *EC*_*prev*_ is slightly better, better, and much better, respectively. Scale 0 indicates that there is no difference between the two methods.

### Statistical analysis

The average grade is used to determine the cleansing quality. For the comparisons between results from *EC*_*prop*_ and those from the other works, two-tailed paired Wilcoxon signed rank tests were applied. Two-tailed *p*-values of <0.05 are considered statistically significant.

## Results

### Clinical evaluation

Five causes of low quality cleansing were reported from the radiologist. The major cause which can be found in the results of *EC*_*prop*_ is the artifacts at T-junctions. For the other four causes, there is no report from the radiologist which means that these are the rear causes and are not often found in CTC images of test data.

The average grade of cleansing quality from 467 test images in Table 3 is 2.89 out of 4. In this cleansing quality, 18.20*%* of the test images are graded as excellent while Table 4 indicates that the most common cause of low quality cleansing is the artifacts at T-junctions which is as high as 69.81*%*.

According to the evaluation results in Table 5, 141 out of 467 or 30.19*%* of the test images are the cases that the artifacts were completely removed and thus the radiologist could not identify any of the remaining artifacts at T-junction in the band view images, whereas the artifacts could be partially removed from 56.75*%* of the test images and the radiologist could still identify the remaining artifacts. Only 13.06*%* of the test images are the cases that none of the artifacts was removed from the test images and the radiologist could identify them all. Sample band view images of the results from *EC*_*syngo*_ that contain complete artifacts at T-junctions are shown in Figs. 7, 8, and 9.

**Table 5:** Amount of the remaining artifacts at T-junctions after applying *EC*_*prop*_

Remaining	0%	1-10%	11-20%	21-30%	31-40%	41-50%	51-99%	100%
Cases	141/467	41/467	30/467	43/467	16/467	45/467	90/467	61/467
Percentage	30.19%	8.78%	6.42%	9.21%	3.43%	9.64%	19.27%	13.06%

The comparison on the cleansing results with 100% remaining artifacts from 13.06*%* (61/467) of test images reveals that *EC*_*prop*_ is more preferable with 9.21*%* while *EC*_*syngo*_ receives only 3.85*%* preferences. The results from Table 6 show that most of the *EC*_*prop*_ results receive slightly better preference much more than those of *EC*_*syngo*_. The comparison between the results from *EC*_*prop*_ and those from *EC*_*syngo*_ shows that the difference is statistically significant (*p*<0.008).

**Table 6:** Comparison on the preference of the cleansing results with 100% remaining artifacts at T-junctions between *EC*_*prop*_ and *EC*_*syngo*_

	1	2	3	Total
*EC*_*syngo*_	14	2	2	18/61(3.85% of 467)
*EC*_*prop*_	38	3	2	43/61(9.21% of 467)

The magnified regions of interest (ROIs) of the axial images in Figs. 7, 8, and 9 are shown at the bottom left. In the first row of Fig. 7, the radiologist evaluates Fig. 7 a and b as perfect cleansing. In the second row of Fig. 7, the blue, red and black arrows point to the artifacts at T-junctions. Without having prior observation in Fig. 7 c and f, the radiologist cannot identify the remaining artifacts at T-junctions in Fig. 7 d while the remaining artifacts at T-junctions in Fig. 7 e can be identified.

The magnified ROI in Fig. 8 b shows the remaining of an AT layer or PV effect layer in the EC result from *EC*_*syngo*_ while there is no AT layer or PV effect layer remains in the EC result from *EC*_*prop*_ as shown in the magnified ROI in Fig. 8 a. For the band view images, the blue, red, and black arrows point to the artifacts at T-junctions. The radiologist cannot identify the artifacts at T-junctions in Fig. 8 d without having prior observation in Fig. 8 c and f. However, the EC results from *E* *C*_*syngo*_ leaves the AT or PV effect layer. Thus, the structure of colonic lumen under the AT layer cannot be seen as shown in Fig. 8 e.

The magnified ROI in Fig. 9 a shows the remaining of a polyp in the EC result from *E* *C*_*prop*_ while there is no remaining of a polyp in the EC result from *EC*_*syngo*_ as shown in the magnified ROI in Fig. 9 b. For the band view images, the blue, red and black arrows point to the remaining artifacts at T-junctions and a polyp. In this case, the radiologist can totally identify the remaining artifacts at T-junctions in the EC result from *EC*_*syngo*_, whereas he cannot identify any artifact in the EC result from *EC*_*prop*_. Moreover, the blue dash circle in Fig. 9 e shows that the polyp is disappeared in the EC result from *EC*_*syngo*_ while *EC*_*prop*_ can preserve it as shown in the red dash circle of Fig. 9 d. Thus, the radiologist rates *EC*_*prop*_ much better than EC method from *EC*_*syngo*_ in this case.

Finally, the degree of fold and polyp preservation is determined by the comparison between the results from *EC*_*prop*_ and those from *EC*_*prev*_ [16]. The comparison results in Table 7 indicate that about 70% of the cleansing results are similar while 18% of the cleansing results indicate that *EC*_*prop*_ is slightly better and 6.21*%* of the cleansing results indicate that *EC*_*prev*_ is slightly better. Moreover, 4.5*%* of the cleansing results indicate that *EC*_*prop*_ is better, while only 0.64*%* of the cleansing results indicate that *EC*_*prev*_ is better. The comparison between the results from *EC*_*prop*_ and those from *EC*_*prev*_ [16] shows that the difference is statistically significant (*p*<0.001).

**Table 7:** Comparison between the results from *E* *C*_*prop*_ and *E* *C*_*prev*_ [16]

EC quality comparison between *E* *C*_*prop*_ and *E* *C*_*prev*_							
	-3	-2	-1	0	1	2	3
#	2/467	3/467	29/467	327/467	84/467	21/467	1/467
%	0.43%	0.64%	6.21%	70.02%	17.99%	4.50%	0.21%

The clinical evaluation shows the improvement of *EC*_*prop*_ over *EC*_*prev*_, and the next subsection will illustrate why *EC*_*prop*_ performs better than *EC*_*prev*_.

### Visual assessment

Visual assessment shows that the proposed AT layer identification is important because it helps *EC*_*prop*_ preserve ATT layers from being removed. An ATT layer can be preserved using the existing methods [13, 19]; however, thin layers [19] or ATT layers [13] between air and FTM are the problem that the existing methods [15, 16] did not take them into consideration.

**Fig. 10:**
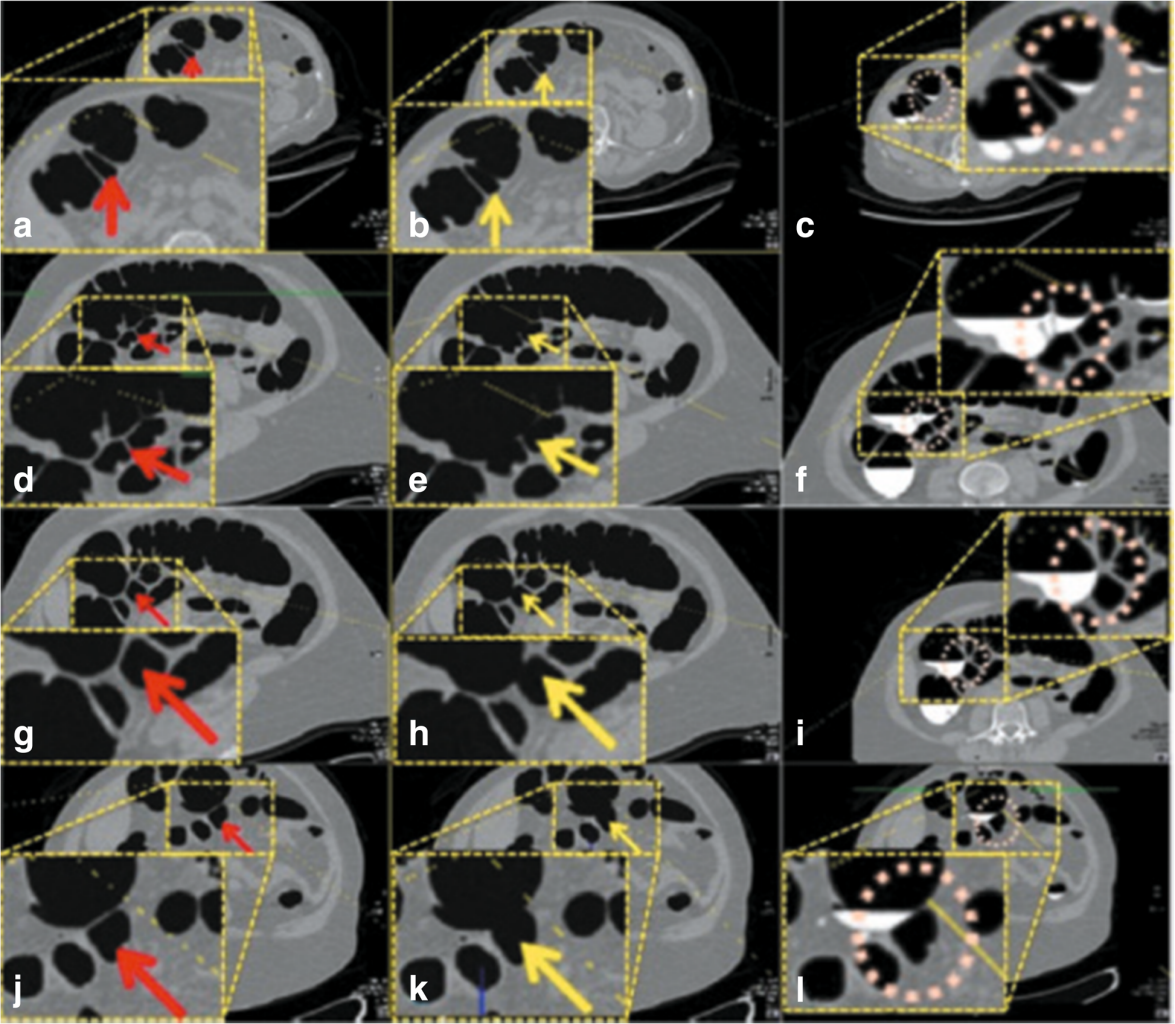
**a**, **d**, **g** and **j** are the axial CTC images of the EC results from *EC*_*prop*_. **b**, **e**, **h** and **k** are the axial CTC images of the EC results from *EC*_*prev*_ [16]. **c**, **f**, **i** and **l** are the axial CTC images without applying any EC

**Fig. 11:**
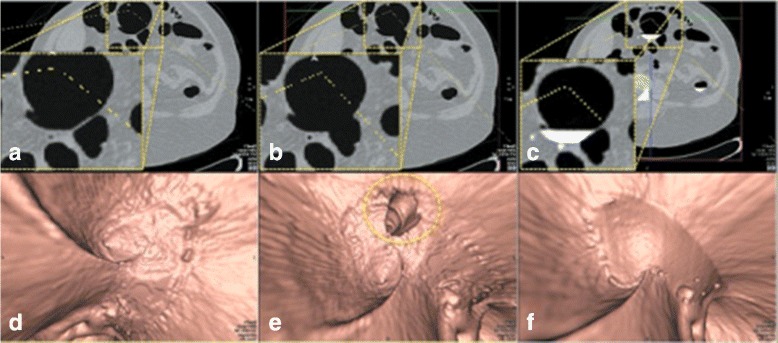
**a** the axial CTC image of the EC result from *EC*_*prop*_. **b** the axial CTC image of the EC result from *EC*_*prev*_ [16]. **c** the axial CTC image without applying any EC. **d** the 3-D band view image of **a**. **e** the 3-D band view image of **b**. **f** the 3-D band view image of **c**

The *EC*_*prev*_ [16] was implemented and was used to compare the results with *EC*_*prop*_. The EC results from *EC*_*prev*_ [16] show that ATT layers are not preserved. The ATT layers are removed after performing *EC*_*prev*_ as shown in the middle column of Fig. 10 while they remain after performing *EC*_*prop*_ as shown in the left column of Fig. 10. For faulty ATT layer removal, the thin soft-tissue layer is disappeared as shown in the axial image of Fig. 11 b and it makes a hole at colonic surface as shown in the band view image in Fig. 11 e.

## Discussion

In this paper, we propose the EC method that uses AT layer identification with integration of GDSD and material fraction model to preserve PEH ST voxels where linear combination of three material fractions is used to remove artifacts at T-junctions, AT layers, and FTM. The AT layer identification is used to distinguish AT layers from ambiguous layers. In order to identify AT layers, the CT scanner table must be at the bottom of a CTC image then we can assume that ATT layers are along the bend of the tagged pool while the orientation of AT layers is in horizontal direction. Next, the numbers of connectivities in horizontal and vertical directions from voxels in an ambiguous layer to voxels in FTM are compared in order to distinguish AT layers from ATT layers. The number of connectivities in horizontal direction is measured to the left and to the right of a voxel while the number of connectivities in vertical direction is measured only downward according to our assumption that an AT layer is over FTM and has orientation in horizontal direction. Thus Algorithm 1 preserves any layer whose orientation is in horizontal direction and removes others from ambiguous layers.

After an AT layer is identified, it is used to find two material fractions from material transition between air and FTM. Moreover, an AT layer is also used to obtain an STT layer which is used to find two material fractions from material transition between STT and FTM. Two material fractions from material transition between air and ST can be found from the mean of CT attenuations of air and the mean of CT attenuations of ST. Then, three pairs of two material fractions can be used to approximate three material fraction of a voxel. Next, GDSD is integrated into a linear combination of two material fractions in Eq. (16) which is used to preserve PEH ST voxels in vicinity of FTM.

The linear combination of three material fractions in Eq. (17) is used to remove the artifacts at T-junctions, AT layers, and FTM where the last term *t*_*TR*_·*L*_*TA*_ is used to remove the influence from the mean of CT attenuations of FTM.

The cleansing quality of the proposed EC method and the amount of the remaining artifacts at T-junctions were evaluated using the radiologist’s opinion. The results show that the number of complete removal of artifacts at T-junction is the highest. Even though, the SA EC method [13] presented the local roughness response to preserve ATT layers, there is no theoretical support that it handles artifacts at T-junctions. Unfortunately, we did not compare *EC*_*prop*_ to SA EC because the computational complexity in theory of solving partial differential equations (PDEs) is higher than that of *EC*_*prev*_. Also, we did not compare the preference between *EC*_*prop*_ and *EC*_*syngo*_ because *EC*_*syngo*_ did not remove AT layers or PV effect layers and could not preserve submerged polyps as shown in Figs. 8 and 9. We only compared our cleansing results to those of *EC*_*syngo*_ for the 100% remaining of artifacts at T-junctions. The comparison on the complete remaining of artifacts at T-junction between the results of *EC*_*prop*_ and those of *EC*_*syngo*_ using the preference from the radiologist shows statistically significant improvement (*p*<0.008).

For the ATT layer preservation results, the middle columns of Figs. 10 and 11 show that ATT layers disappeared after using *EC*_*prev*_, while the left columns show that ATT layers are preseved after using *EC*_*prop*_. The reason is that the rut-enhancement function [13, 15, 16] is designed for enhancing submerged rut-like structure such as submerged thin folds but not ATT layers. On the other hand, the proposed AT layer identification can exclude ATT layers from being removed during EC by changing CT attenuations into ST range using Eq. (16). The comparison between the results of *EC*_*prop*_ and *EC*_*prev*_ [16] using the preference from the radiologist also shows statistically significant improvement (*p*<0.001).

**Fig. 12:**
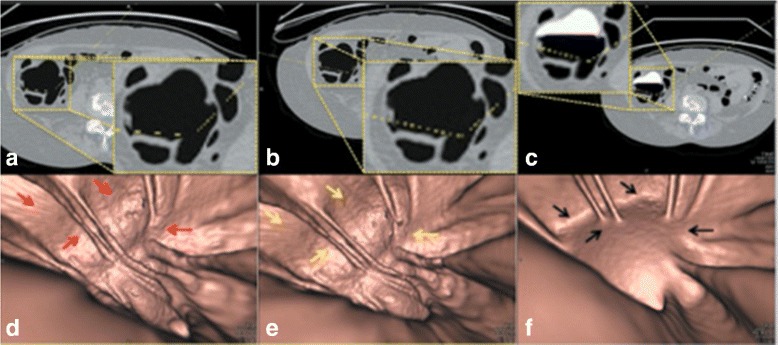
**a** the axial CTC image of the EC result from *EC*_*prop*_. **b** the axial CTC image of the EC result from *EC*_*prev*_ [16]. **c** the axial CTC image without applying any EC. **d** the 3-D band view image of **a**. **e** the 3-D band view image of **b**. **f** the 3-D band view image of **c**

For most of the cases, the radiologist concluded that the EC results from *EC*_*prop*_ and those from *EC*_*prev*_ are about the same. Figure 12 shows the band view images where the red, yellow, and black arrows point at the artifacts at T-junctions but the radiologist cannot identify the remaining artifacts at T-junctions after performing EC in both Fig. 12 d and e without having prior observation from Fig. 12 c and f.

For polyp detection in 10 cases (20 scans) from King Chulalongkorn Memorial hospital, there are 17 polyps which can be divided into two groups: a group of 12 polyps with size ≥6 mm and a group of five polyps with size ≥10 mm, respectively. For partially or completely submerged polyps in FTM, there are nine polyps with size ≥6 mm and three polyps with size ≥10 mm, respectively. All of them are visible in *EC*_*prop*_ and *EC*_*prev*_ where they could hardly be found in 3-D band view images before applying EC methods. Thus, the sensitivity for polyp detection after EC is as high as 100% (17/17) for both *EC*_*prop*_ and *EC*_*prev*_.

The time complexity of *EC*_*prev*_ is *O*(log*N*) for both linear combination of triple arch projections of three-material modeling and two-material transition projection. For complexity of *EC*_*prop*_, the proposed lung and bone removal, AT layer identification and GDSD approximation consume *O*(*N*) where the rest of *EC*_*prop*_ takes as much time as *EC*_*prev*_. The structural analysis (SA) EC method [13] uses the level set method as the core of segmentation part. It has been pointed out by Adalsteinsson et al. [38] that the computational complexity of level set for the three space dimensions is *O*(*N* ^3^) per time step. Thus, the time complexity of *EC*_*prop*_ is in between *EC*_*prev*_ and the structural analysis (SA) EC method [13]. Time complexity is approximately three minutes per scan of a patient when it is executed in OS X EL Capitan version 10.11.16 with processor 2.7 GHz intel Core i5 and memory 8 GB of MacBook Pro.

## Conclusion

We propose the EC in CTC images using AT layer identification with integration of gradient directional second derivative and material fraction model. The proposed EC method can identify and distinguish AT layers from ATT layers using the proposed AT layer identification. The integration of the gradient directional second derivative into linear combination of two material fractions of material transition between ST and FTM is proposed in order to preserve submerged PEH ST voxels in FTM, while ATT layers can also be preserved. The artifacts at T-junctions, FTM, and AT layers can be removed successfully by using linear combination of three material fractions. To confirm the efficiency of the proposed EC method, the clinical evaluation was performed by the radiologist and the average grade of cleansing quality is 2.89 out of 4. The proposed EC method was compared to the syngo.via commercial software from Siemens CT scanner and the fast three-material fraction model. The comparison results show that the radiologist prefers the proposed EC method over the syngo.via commercial software and the fast three-material model with statistical significance.

## Abbreviations

ADC: Adaptive PEH correction method
AT: Air-tagging or the artifact layer between air and fecal-tagging material
ATT: Air-tissue-tagging or thin soft tissue between air and fecal-tagging material
CT: Computed tomography
CTC: Computed tomography colonography
EC: Electronic cleansing
*EC*_*prev*_: The fast three-material modeling EC method
*EC*_*prop*_: The proposed EC method
*EC*_*syngo*_: The syngo.via Client 3.0 commercial software from Siemens CT Scanner
FTM: Fecal-tagging material
GDSD: Gradient directional second derivative
HU: Hounsfield unit
PDEs: Partial differential equations
PEH: Pseudo-enhancement
ROI: Region of interest
SA: Structural analysis EC method
ST: Soft tissue
STT: The boundary between soft tissue and fecal-tagging material
VC: Virtual colonography
WRAMC: Walter Reed Army Medical Center

## References

[1] Coin CG, Wollett FC, Coin JT, Rowland M, Deramos RK, Dandrea R (1983). Computerized radiology of the colon: A potential screening technique. Comput Radiol.

[2] Shigeru LH, Muraki S, Kaufman A, Bartz D, He T (1997). Virtual voyage: Interactive naviation in the human colon. Proc. ACM SIGGRAPH..

[3] Ferrucci JT (2001). Colon cancer screening with virtual colonoscopy:promise, polyps, politics. Amer J Roentgenol.

[4] Morrin MM, LaMont JT (2003). Screening virtual colonoscopy-ready for prime time?. N Engl J Med.

[5] Kaufman AE, Lakare S, Kreeger K, Bitter I (2005). Virtual colonscopy. Commun ACM.

[6] Society AC (2013). Cancer Facts & Figures.

[7] Institute ITDNC (2015). Hospital-Based Cancer Registry.

[8] Hong L, Liang Z, Visawambharant A, Kaufman A, Wax M (1997). Reconstruction and visualization of 3d models of colonic surface. IEEE Trans Nucl Sci.

[9] Serlie I, Vos F, Stoker J, Truyen R, Gerritsen F, Nio Y, Post F. Improved visualization in virtual colonoscopy using image-based rendering. In: Proc IEEE Joint Eurographics TCVG Symp Vis. IEEE Computer Society: 2001. p. 137–46.

[10] Hara AK, Johnson CD, Reed JE, Ahlquist DAA, Nelson H, MacCarty RL (1997). Detection of colorectal polyps with ct colonography: Initial assessment of sensitivity and specificity. Radiology.

[11] Fenlon HM, Nunes DP, Schroy PC, Barish MA, Clarke PD, Ferrucci JT (1999). A comparison of virtual and conventonal colonoscopy for the detection of colorectal polyps. N Engl J Med.

[12] Yee J, Akerkar GA, Hung RK, Steinauer-Gebauer AM, Wall SD, McQuaid KR (2001). Colorectal neoplasia: Performance characteristics of ct colonography for detection in 300 patients. Radiology.

[13] Cai W, Zalis ME, Näppi J, Harris GJ, Yoshida H (2008). Structre-analysis method for eletronic cleansing in cathartic and noncathartic ct colonography. Meds Phys.

[14] Cai W, JG L, Zalis EM, Yoshida H (2010). Mosaic decomposition: An electronic cleansing method for inhomogeneously tagged regions in noncathartic ct colonography. IEEE Trans Med Imaging.

[15] Lee H, Kim B, Lee J, Kim SH, Shin Y, Kim T (2013). Fold-preserving electronic cleansing using a reconstruction model integrating material fractions and structural responses. IEEE Trans Biomed Eng.

[16] Lee H, Lee J, Kim B, Kim SH, Shin Y (2014). Fast three-material modeling with triple arch projection for electronic cleansing in ctc. IEEE Trans Biomed Eng.

[17] Linguraru MG, Panjwani N, Fletcher JG, Summers RM (2011). Automated image-based colon cleansing for laxative-free ct colonography computer-aided polyp detection. Med Phys.

[18] Wang S, Li L, Cohen H, Mankes S, Chen JJ, Liang Z (2008). An em approach to map solution of segmenting tissue mixture percentages with application to ct-based virtual colonoscopy. Med Phys.

[19] Ravestejin VVF, Vos FM, Serlie IWO, Truyen R, van LJ V (2008). Thin layer tissue classification for electronic cleansing of ct colonography data. ICPR 2008, 19th International Conference.

[20] Serlie IWO, Vos FM, Truyen R, Post FH, Stoker J, Vliet LJV (2010). Electronic cleansing for computed tomography(ct) colonography using a scale-invariant three-material model. IEEE Trans Biomed Eng.

[21] Ravestejin VVF, Boellaard TN, Paardt VDMP, Serlie WOI, Haan CDM, Stoker J, Vliet JVL, Vos FM (2013). Electronic cleansing for 24-h limited bowel preparation ct colonography using principal curvature flow. IEEE Trans Bio Med Eng.

[22] Lefere PA, Gryspeerdt SS, Dewspelaere J, Baekelandt M, Holsbeeck BGV (2002). Dietary fecal tagging as a cleansing method before ct colonography: Initial result-polyp detection and patient acceptance. Radiology.

[23] Zalis ME, Blake MA, Cai W (2012). Diagnostic accuracy of laxative-free computed tomographic colonography for detection of adenomatous polyps in asymtomatic adults: a prospective evaluation. Ann Intern Med.

[24] Summers RM (2009). The elephant in the room: Bowel preparation for ct colonography. Acad Radiol.

[25] Pickhardt PJ, Hassan C, Halligan S, Marmo R (2011). Colorectal cancer: ct colonography and colonoscopy for detection-systematic review and meta-analysis. Radiology.

[26] Nagata K, Endo S, Honda T, Yasuda T, Hirayama M, Takahashi S, Kato T, Horita S, Furuya K, Kasai K, Matsumoto H, Kimura Y, Utano K, Suginoto H, Kato H, Yamada R, Yamamichi J, Shimamoto T, Ryu Y, Matsui O, Kondo H, Doi A, Abe T, Yamano OH, Takeuchi K, Hanai H, Saida Y, Fukuda K, Näppi J, Yoshida H, AGAF, FACG (2016). Accuracy of ct colonography for detection of polypoid and nonpolypoid neoplasia by gastroenterologists and radiologists: A nationawide multicenter study in japan. Am J Gastroenterol.

[27] Colorectal Cancer Prevention and Screening. http://www.uwhealth.org/coloncancerscreening/virtual-colonoscopy/29527. Accessed 31 Aug 2017.

[28] Prepare for a Virtual CT Colonoscopy scan. San Francisco. http://radiology.ucsf.edu/patient-care/prepare/vc. Accessed 31 Aug 2017.

[29] Virtual Colonoscopy (CT Scan). Bangkok, Thailand. https://www.bumrungrad.com/en/digestive-diseases-gi-center-treatment-bangkok-thailand/procedures/virtual-colonoscopy-ct-scan. Accessed 31 Aug 2017.

[30] Näppi J, Yoshida H (2008). Adaptive correction of the pseudo-enhancement of ct attenuation for fecal-tagging ct colonography. Med Image Anal.

[31] Tsagaan B, Näppi J, Yoshida H (2009). Nonlinear regression-based method for pseudoenhancement correction in ct colonography. Med Phys.

[32] Liu J, Yao J, Summers RM (2008). Scale-based scatter correction for computer-aided polyp detection in ct colonography. Med Phys.

[33] Chen Y, Zhang Y, Yang J, Cao Q, Yang G, Chen J, Huazhong S, Luo L, Coatrieux J, Feng Q (2016). Curve-like structure extraction using minimal path propagation with backtracking. IEEE Trans Image Proc.

[34] Serlie IWO, Vos FM, Truyen R, Post FH, Vliet LJV (2007). Classifying ct image data into material fractions by a scale and rotation invariant edge model. IEEE Trans Image Process.

[35] Zhang Y, Zhang Y, Lv YD, Hou XX, Liu FY, Jia WJ, Yang MM, Phillips P, Wang SH. Alcoholism detection by medical robots based on hu moment invariants and predator-prey adaptive-inertia chaotic particle swarm optimization. IEEE Trans Image Proc. 2017. doi:10.1016/j.compeleceng.2017.04.009.

[36] Chen Y, Yang J, Zhang Y, Huazhong S, Luo L, Coatrieux J, Feng Q. Structure-adaptive fuzzy estimation for random-value impulse noise suppression. IEEE Trans Circ Syst Video Technol. 2016. doi:10.1109/TCSVT.2016.2615444.

[37] Pickhardt PJ, Choi JR, Hwang I, Butler JA, Puckett ML, Hildebrandt H, K WR, Nugent PA, Mysliwiec PA, Schindler WR (2003). Computer tomographic vitual colonoscopy to screen for colorectal neoplasia in asymptomatic adults. N Engl J Med.

[38] Adalsteinsson D, Sethian JA (1995). A fast level set method for propagating interfaces. J Comput Phys.

